# A New Geldanamycin Analogue from *Streptomyces hygroscopicus*

**DOI:** 10.3390/molecules15031161

**Published:** 2010-03-03

**Authors:** Hao Zhang, Guang-Zhi Sun, Xiang Li, Hong-Yu Pan, Yan-Sheng Zhang

**Affiliations:** 1 School of Resources and Environment, Jilin Agricultural University, Changchun, 130118, China; E-Mail: haozhang100@163.com (H.Z.); 2 Institute of Agricultural Modernization, Jilin Agricultural University, Changchun, 130118, China; E-Mail: gzsun1967@yahoo.com (G.-Z.S.); 3 College of Plant Science, Jilin University, Changchun, 130062, China; E-Mail: drxiang@hotmail.com (X.L.); 4 Wuhan Botanical Garden, Chinese Academy of Sciences, Wuhan, 430074, China

**Keywords:** *Streptomyces hygroscopicus*, 11-methoxy-17-formyl-17-demethoxy-18-*O*-21-*O*-dihydrogeldanamycin, geldanamycin

## Abstract

A new geldanamycin analogue was isolated from *Streptomyces hygroscopicus* A070101. The structure was elucidated as 11-methoxy-17-formyl-17-demethoxy-18-*O*-21-*O*-dihydrogeldanamycin (**1**) on the basis of extensive 1D and 2D NMR as well as HRESI-MS spectroscopic data analysis. Compound **1 **showed considerable cytotoxicity (SRB) against human cancer cell lines (breast cancer MCF-7, skin melanoma SK-MEL-2 and lung carcinoma COR-L23).

## Introduction

**Figure 1:**
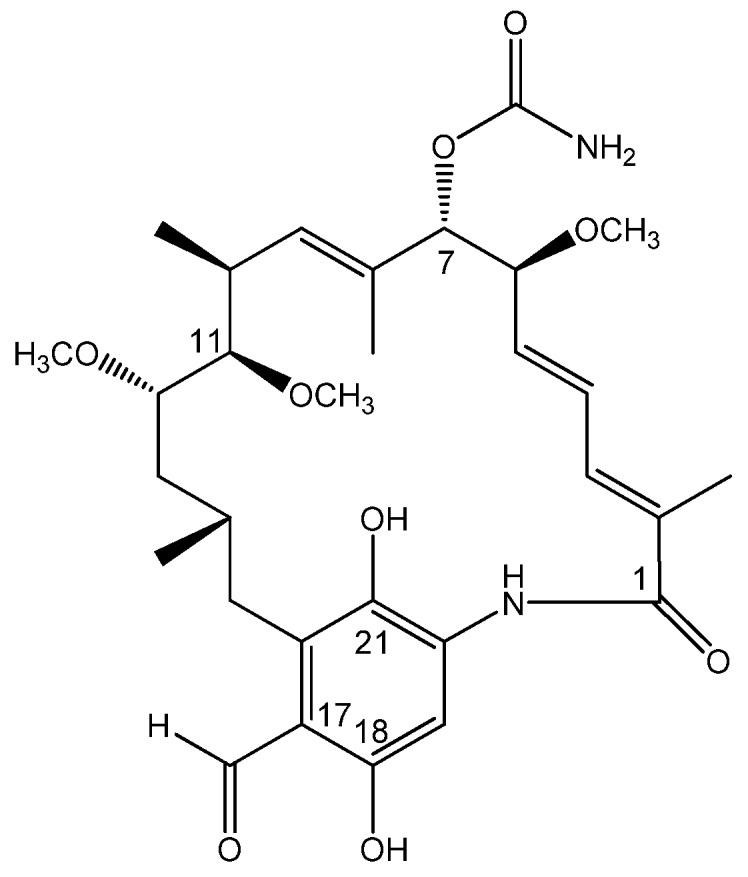
Structure of 11-methoxy-17-formyl-17-demethoxy-18-*O*-21-*O*-dihydrogeldanamycin (**1**).

Steroid hormone receptors are generally intracellular receptors and initiate signal transduction for steroid which lead to changes in gene expression over a time period ranging from hours to days [1]. During the translation, steroid receptors are assembled into a multi-protein complex containing hsp90 (one of the most abundant proteins expressed in cells) [2], p23, an immunophilin, and often some hsp70 [3]. Geldanamycin analogues were found to bind to hsp90 and disrupt its function, impedes dexamethasone-dependent trafficking of the glucocorticoid receptor from the cytoplasm to the nucleus, led many of them are protooncogenic and play a prominent role in cancer [4]. For example, geldanamycin and its analog 17-AAG showed significant cytotoxicity against human cancer cell line SKBr3 (IC_50_ = 41 and 33 nM, respectively) [5]. During our continued work on bioactive bacteria and fungi, a *Streptomyces hygroscopicus* strain was isolated from the soil of Chang-Bai Mountain. A new geldanamycin analogue was isolated from a 20 L fermentation of this strain. Its structure was elucidated as 11-methoxy-17-formyl-17-demethoxy-18-*O*-21-*O*-dihydrogeldanamycin (**1**, Figure 1) on the basis of extensive 1D and 2D NMR as well as HRESI-MS spectroscopic data analysis. Compound **1 **was tested with five-day *in vitro* SRB cytotoxicity against human tumors cell lines and showed considerable cytotoxicity against human cancer cell lines (breast cancer MCF-7, skin melanoma SK-MEL-2 and lung carcinoma COR-L23).

## Results and Discussion

### Characterization of compound ***1***

**Figure 2:**
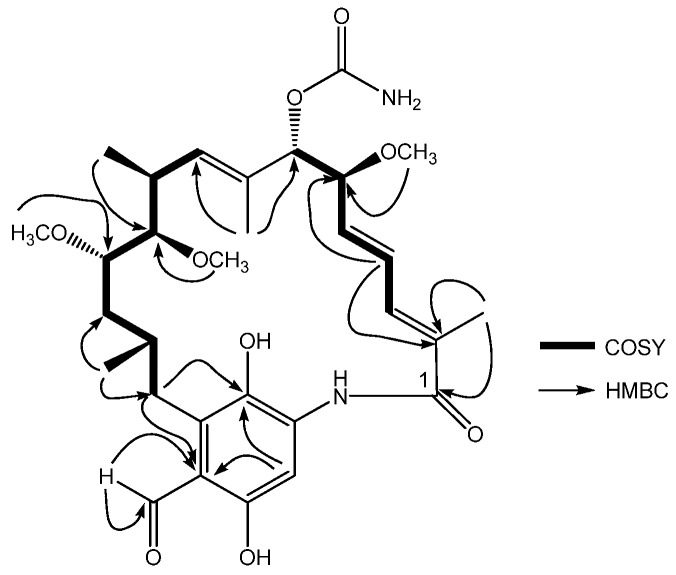
COSY and HMBC of compound **1**.

The molecular formula of compound **1** was determined as C_30_H_42_N_2_O_9_ on the basis of its HR-ESI-MS (*m/z* 575.2981 [M+H]^+^, calcd. 575.2969) and NMR data (Table 1). Analysis of the ^13^C-NMR spectrum and DEPT experiments, allowed the identification of seven methyl groups, two methylenes, eleven methines and ten quaternary carbons. Analysis of the ^1^H-^1^H COSY (Figure 2) and HMQC spectra suggested the presence of two ^1^H-^1^H spin systems: H_3_-H_4_- H_5_-H_6_-H_7_, H_9_-H_10_-H_11_-H_12_-H_13_-H_14_-H_15_. The chemical shifts of the protons and carbons (Table 1) were similar to those of the previously reported compound, 17-formyl-17-demethoxy-18-*O*-21-*O*-dihydrogeldanamycin, a geldanamycin analogue isolated from recombinant *S. hygroscopicus* strain [6]. The main differences between the two metabolites concerned a newly appeared methoxyl group [δ_C_ 57.2, δ_H_ 3.37 (*s*)] in compound **1**. The location of the methoxyl group was established taking into account the correlation observed between 11-OCH_3_ (δ 3.37) and C-11 (δ 156.8) in the HMBC experiment of **1** (Figure 2). The coupling constant between the H5 and H6 (11.2 Hz), H9 and H10 (8.8 Hz), were similar to the literature values 10.4 Hz and 9.6 Hz [6], respectively, led to determine the relative stereochemistry same as reported compound KOSN 1645.

**Table 1:** The NMR data of compound **1**.^a^

No.	Compound 1			KOSN 1645 ^b^	
	^1^H-NMR	^13^C-NMR	DEPT	^1^H-NMR	^13^C-NMR
1	-	169.1	C	-	167.3
2	-	135.2	C	-	136.7
3	7.01 (*d*, *J* = 12.0)	124.3	CH	6.85 (*d*, *J* = 11.2)	124.7
4	6.22 (*t*, *J* = 12.0)	128.6	CH	6.35 (*t*, *J* = 11.2)	127.1
5	5.24 (*t*, *J* = 11.2)	133.3	CH	5.71 (*t*, *J* = 10.4)	132.9
6	3.99 (*d*, *J* = 11.2)	79.6	CH	4.31 (*d*, *J* = 10.4)	80.9
7	4.83 (*s*)	80.7	CH	4.96 (*s*)	82.8
8	-	132.6	C	-	133.4
9	5.47 (*d*, *J* = 8.8)	133.1	CH	5.94 (*d*, *J* = 9.6)	133.4
10	2.51 (*m*)	32.1	C	2.81 (*qn*, *J* = 7.6)	32.3
11	3.73 (*m*)	72.9	CH	3.63 (*m*)	74.7
12	3.57 (*m*)	79.3	CH	3.5 (*m*)	80.3
13	1.86 (*m*)	33.7	CH_2_	1.88 (*br*)	35.4
14	1.83 (*m*)	29.3	CH	1.6-1.8 (*ov*)	29.2
15	2.58 (*m*)	33.1	CH_2_	2.72 (*br*)	33.4
16	-	126.9	C	-	127.3
17	-	114.2	C	-	113.1
18	-	159.3	C	-	159.4
19	7.71 (*s*)	105.6	CH	7.93 (*s*)	104.7
20	-	132.3	C	-	135.6
21	-	140.6	C	-	136.4
2-Me	1.65 (*s*)	11.8	CH_3_	1.59 (*s*)	12.4
8-Me	1.68 (*s*)	11.3	CH_3_	1.79 (*s*)	12.6
10-Me	0.91 (*d*, *J* = 7.2)	10.9	CH_3_	0.93 (*d*, *J* = 7.2)	12.1
14-Me	0.97 (*d*, *J* = 8.8)	23.1	CH_3_	0.95 (*d*, *J* = 8.8)	22.4
6-OMe	3.29 (*s*)	55.9	CH_3_	3.36 (*s*)	56.9
11-OMe	3.37 (*s*)	57.2	CH_3_	-	-
12-OMe	3.31 (*s*)	57.7	CH_3_	3.22 (*d*)	57.1
17-CHO	9.81 (*s*)	191.0	C	10.09 (*s*)	193.2
7-carbamate	8.50 (*s*)	155.5	C	8.73 (*s*)	156.5

^a^ Compound **1 **was measured in DMSO-*d*6 and chemical shifts are expressed in ppm; ^b^^1^H and ^13^C-NMR.

data (in CDCl_3_) of KOSN 1645 were extracted from [6].

**Figure 3:**
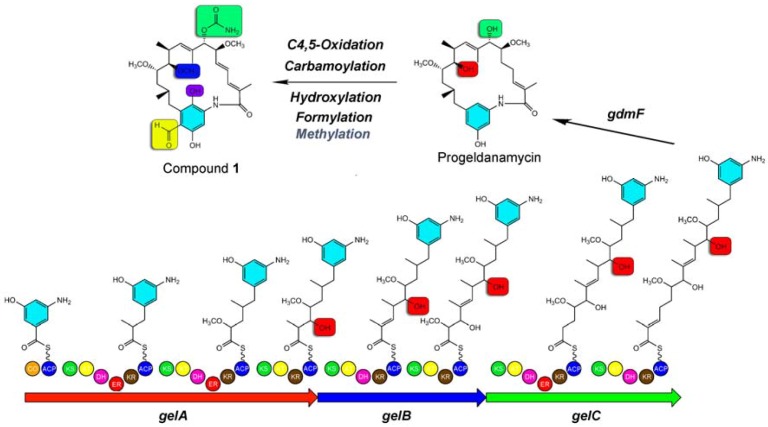
Speculated biosynthetic pathway of compound **1**.

The biosyntheses of geldanamycin analogues were reported to be involved in the assembly of 3-amino-5-hydroxybenzoic acid (AHBA) as a starter unit, following elongation with the acyl-Coenzyme A substrates malonyl-CoA, methylmalonyl-CoA, and 2-methoxymalonyl-ACP, the polyketide intermediate undergoes intra-molecular lactamization by gdmF to form progeldanamycin [5,7] (Figure 3). The compound **1** isolated in this study and previously reported herbimycin A [6], proposed that an *O*-methylation step exist after the formation of polyketide backbone which may lead to identify a new *O*-methyltransferase. To prove this hypothesis, mutant lines could be established for screening of this 11-*O*-methyltransferase.

### Bioactivity results

Compound **1** was tested in anti-tumor bioassays and showed significant cytotoxicity (SRB) against several human cancer cell lines: breast cancer MCF-7 (IC_50_ = 142 nM), skin melanoma SK-MEL-2 (IC_50_ = 496 nM) and lung carcinoma COR-L23 (IC_50_ = 278 nM). 

## Conclusions

In summary, we have isolated a new geldanamycin analogue 11-methoxy-17-formyl-17-demethoxy-18-*O*-21-*O*-dihydrogeldanamycin (1) from a 20 L of *Streptomyces hygroscopicus* A070101 fermentation broth. Compound 1 showed considerable cytotoxicity against human cancer cell lines (breast cancer MCF-7, skin melanoma SK-MEL-2 and lung carcinoma COR-L23).

## Experimental

### General

The ^1^H- and ^13^C-NMR spectra were measured on a Bruker Avance DRX 500 NMR spectrometer in DMSO-*d*_6_, using TMS as an internal standard. Chemical shifts (*δ*) are expressed in parts per million (ppm), with the coupling constants (*J*) reported in Hertz (Hz). The HR-ESI mass spectrum was obtained from a MDS SCIEX API QSTAR-MS instrument. TLC was performed with silica gel plates (Macherey -Nagel, SilG / UV254, 0.20mm); Semi-preparative HPLC was carried out with Agilent 1100 on a Zorbax C_18_ column (250 x 10 mm, Phenomenex, Torrance, CA), UV absorption data (λ_280_) were analyzed with Agilent Chemstation Ver 8.01. All solvents used in this study were HPLC grade, purchased from the Chinese Chemical Group, Beijing, China. 

### Bacterial strain and growth conditions

Bacterial strain A070101 was obtained from Chang-Bai Mountain soil during a systematic screening of ginsenoside-glucosidase-produced bacteria [10] and was identified as *Streptomyces hygroscopicus* by professor Zhao-Yang He (Jilin Agricultural University). Geldanamycin production medium (GPM), consist of sucrose (50 g/liter), peptone (2 g/liter), tryptone (2 g/liter), yeast-extract (2 g/liter), Gerber's oatmeal (5 g/liter), and Brer Rabbit molasses (10 ml/liter) (pH = 7.0), was used to produce geldanamycin homologues [11]. The fermentation was carried in fifty 1 L flasks at 28 °C for 7 days.

### Extraction and isolation of compound ***1***

The lyophilized culture broth was extracted with 80% EtOH at room temperature. The extract was concentrated under reduced pressure to give the pale brown residue (625 mg) that was fractionated by reverse phase (C-8) chromatography using H_2_O, aqueous MeOH (30%, 60%, 90%) and MeOH to give four fractions: the 90% MeOH fraction was further fractionated on a Sephadex LH- 20 column (CHCl_3_: MeOH=1:1) and then purify by semi-preparative HPLC to yield compound **1** (3.6 mg, 0.18 mg/L, whereas yield of geldanamycin was reported as ~10 mg/liter) [6].

### In vitro cytotoxicity assays

Five-day *in vitro* SRB cytotoxicity tests against human tumors cell lines were carried out at the Cell Culture Laboratory, Pharmaceutical College, Jilin University, using modified protocols for MCF-7 (breast cancer), SK-MEL-2 (skin melanoma) and COR-L23 (lung carcinoma), the normal cells were used as control [12]. Generally, 5x10^3^/mL cells were placed in a 24-well plate and treated with compound **1**. The plate was incubated at 37 °C for 5 days. Then the medium was removed from the 24-well plate, and 10% ice-cold TCA (trichloroacetic acid, 1 mL) was added. The plate was kept at 4 °C for two hours after which was washed four times with cold water, then stained with SRB (Sulforhodamine B, Sigma St. Louis, MO, USA). After washing with 1% acetic acid, the bound dye was solubilized with Tris base A (Sigma) and 100 μL of each sample were transferred into a 96-well plate, and then read at 492 nm.
